# Amphetamine, but not methylphenidate, increases ethanol intake in adolescent male, but not in female, rats

**DOI:** 10.1002/brb3.939

**Published:** 2018-02-19

**Authors:** Paul Ruiz, Aldo Calliari, Patricia Genovese, Cecilia Scorza, Ricardo Marcos Pautassi

**Affiliations:** ^1^ Instituto de Investigación Médica M. y M. Ferreyra (INIMEC—CONICET‐Universidad Nacional de Córdoba) Córdoba Argentina; ^2^ Facultad de Veterinaria Universidad de la República Montevideo Uruguay; ^3^ Departmento de Neurofarmacología Experimental Instituto de Investigaciones Biológicas Clemente Estable Montevideo Uruguay; ^4^ Facultad de Psicología Universidad Nacional de Córdoba Córdoba Argentina

**Keywords:** adolescence, amphetamine, ethanol, methylphenidate, motor behavior

## Abstract

**Introduction:**

There has been an increasing interest in analyzing the interactions between stimulants and ethanol during childhood and adolescence. Stimulants are used to treat attention‐deficit hyperactivity disorder (ADHD) in these developmental stages, during which ethanol initiation and escalation often occur.

**Methods:**

This study assessed the effects of repeated d‐amphetamine (AMPH) or methylphenidate (MPH) treatment during adolescence [male and female Wistar rats, between postnatal day (PD) 28 to PD34, approximately] on the initiation of ethanol intake during a later section of adolescence (PD35 to PD40).

**Results:**

Amphetamine and MPH exerted reliable acute motor stimulant effects, but there was no indication of sensitized motor or anxiety responses. MPH did not affect dopamine (DA) levels, whereas AMPH significantly reduced insular levels of DA in both sexes and norepinephrine levels in females only. Repeated treatment with AMPH, but not with MPH, enhanced ethanol intake during late adolescence in male, but not in female, rats.

**Conclusion:**

A short treatment with AMPH during adolescence significantly altered DA levels in the insula, both in male and females, and significantly enhanced ethanol intake in males. The present results suggest that, in adolescent males, a very brief history of AMPH exposure can facilitate the initiation of ethanol intake.

## 1 INTRODUCTION

Worldwide, up to 5% of children and youth up to 18 years old are diagnosed with attention‐deficit hyperactivity disorder (ADHD). This syndrome affects attention and school performance, activity levels, and is mostly treated with psychostimulants [e.g., d‐amphetamine (AMPH) and methylphenidate (MPH)].

Despite recent studies suggesting the relative advantages of the use of stimulants as treatment for ADHD (Dalsgaard, Kvist, Leckman, Nielsen, & Simonsen, 2014), yet see earlier work by Lambert and Hartsough (1998), there is still an ongoing discussion about the effects of these drugs upon the patient's risk of development of ethanol‐related or other drug‐related problems (Quinn et al., 2017). Late infancy (Pilatti, Godoy, Brussino, & Pautassi, 2013) and adolescence (Pilatti, Read, & Pautassi, 2017) are developmental stages in which ethanol initiation normatively occurs and in which treatments that alter developmental trajectories of dopamine (DA) or other transmitter systems are likely to facilitate the escalation toward ethanol abuse or dependence (Pascual, Boix, Felipo, & Guerri, 2009). AMPH and MPH exert their action by increasing the synaptic levels of catecholamine transmitters, such as DA, either by increasing their release from or by inhibiting their reuptake into neurons.

In other words, a significant fraction of children and adolescent are concurrently or, more likely, sequentially exposed to stimulants and ethanol and more information is needed on how repeated exposure to the former drugs affect the initiation of ethanol intake and preference (Mannuzza et al., 2008). Specifically, treatment with stimulants begins at around age 9–11 (Mannuzza et al., 2008), approximately, whereas age of ethanol initiation (i.e., first intake of a full standard drink) takes place between 14 and 16 years of age (Pilatti et al., 2017).

Animal studies suggest that repeated stimulant exposure may sensitize the neural and behavioral response to subsequent drug challenges, enhancing the reinforcing effects of these drugs and rendering the individual at risk for drug addiction (Mannuzza et al., 2008; Schenk & Davidson, 1998). The sensitization induced by drugs of abuse, which is thought to reflect the transition from a nonaddicted to an addicted state, can occur at many levels, yet it has been most often analyzed by assessing the progressive increase in drug‐induced motor stimulation that results from repeated drug exposure (Camarini & Pautassi, 2016). It should be noted, however, that some have suggested that a low level of response to the stimulant effects of ethanol or psychostimulants can also be a risk factor for drug addiction disorders. A blunted response to ethanol may promote ethanol initiation or escalation (Quinn & Fromme, 2011; Schuckit et al., 2000), for instance by facilitating binge drinking to reach a preferred level of intoxication (Schuckit, 2009). Furthermore, rats classified as low cocaine responders—based on their acute locomotor response to cocaine—exhibited greater cocaine‐induced behavioral sensitization and greater operant responding for cocaine (Mandt, Schenk, Zahniser, & Allen, 2008).

It is difficult to define the boundaries of adolescence in rodents, yet a seminal review defined the period as encompassing postnatal days 28–42 [PDs 28–42; (Spear, 2000)]. This conservative definition is still used [see (McClintick et al., 2015)], yet further refinements have been made, with some denominating the PD 28–42 stage as mid‐adolescence or early adolescence (Doremus‐Fitzwater & Spear, 2016; Karanikas, Lu, & Richardson, 2013), the preceding week (PDs 21–28, sometimes up to PD34) as juvenile period, and the period between PD46 and PD 59 as late adolescence (Burke & Miczek, 2014) or emerging adulthood.

The few preclinical studies that, to our knowledge, dealt with the relationship between ADHD treatments and subsequent ethanol intake have yielded inconsistent results, and none conducted both events within the adolescent stage of development. Gill, Chappell, Beveridge, Porrino, and Weiner (2014) found greater drinking of sweetened ethanol after a lengthy MPH treatment in young adult, male, rats reared under environmental enrichment, but not in those reared under standard or isolated housing conditions. Earlier work (Vendruscolo, Izídio, Takahashi, & Ramos, 2008) found greater ethanol intake in adult female, but not in male, spontaneously hypertensive (SHR) rats that had been given MPH (2.0 mg/kg, twice daily during 16 days) during adolescence. A lack of effect of MPH [treatment during PDs 21–35] upon ethanol consumption of SHR rats was reported by Soeters, Howells, and Russell (2008). These studies have the caveats of either testing ethanol intake only in one sex, with ethanol mixed in sugar or they conducted the administration of the stimulant in one developmental stage (i.e., adolescence), yet tested for ethanol intake at other stage (i.e., adulthood). These “serial” designs are valuable to understand the lingering, persistent consequences of early stimulant treatment, yet they do not mirror well the type of exposure that takes place in humans. As described, age at initiation of treatment for ADHD precedes that of ethanol initiation, yet both take place well before adulthood and within the stages of late infancy and adolescence.

More work is needed to ascertain ethanol use liability in adolescents treated with stimulants. Moreover, sex differences should be more carefully pursued. Males and females differentially react to drugs (Wille‐Bille et al., 2017), yet despite the increasing awareness of these differences and the explicit suggestion to have same‐sex representativeness (McCullough et al., 2014), women/female are still a neglected group in epidemiological and preclinical research. Last but not least, repeated MPH exposure and AMPH exposure are also used to model manic and bipolar conditions in laboratory animals (Frey et al., 2006), and there is some evidence suggesting exacerbated ethanol consumption in individuals with bipolar disorders (Strakowski & DelBello, 2000).

The main aim of this study was to assess, in Wistar male and female adolescent rats, the effects of repeated MPH or AMPH treatment (between PD28 to PD34, approximately) on the acquisition of voluntary ethanol drinking during PD36 to PD42. Motor activity patterns and anxiety response [a promoter of ethanol drinking in adolescent rats (Acevedo, Fabio, Fernandez, & Pautassi, 2016)] induced by the repeated drug treatment were also assessed.

Specifically, in Experiment 1 of this study, the rats received daily administration of MPH or vehicle, during 4 days, with motor activity measured after each administration and then were tested for ethanol intake in an intermittent‐access intake protocol. MPH did not induce sensitization to the motor effects of the drug nor significantly affected ethanol intake patterns. In Experiment 2, we analyzed ethanol intake in male and female adolescents after an induction‐expression sensitization protocol with AMPH. In this protocol, animals are given repeated treatment with a drug or its vehicle followed by a challenge test, in which all receive a lower dose of the drug than that used during training. The rationale is that the acute effect of the lower dose will be higher (i.e., sensitized) in those with chronic exposure to the drug than in drug‐naïve controls (Camarini & Pautassi, 2016; Kuczenski & Segal, 2001).

We also measured insular levels of dopamine (DA) after MPH treatment, whereas DA, norepinephrine, and serotonin (5‐HT) levels at the insular cortex were measured after AMPH treatment. DA, norepinephrine, and 5‐HT are implicated in the therapeutic effects of MPH and AMPH (Kuczenski & Segal, 2001), yet sustained treatment with stimulants can induce neuroadaptive changes in these systems (Cryan, Hoyer, & Markou, 2003). These effects are found across several brain areas including mesolimbic and mesocortical pathways. Based on previous studies, however, we decided to focus on the insular cortex. We have recently observed that low levels of dopamine at the insula were associated, in adolescent Wistar rats, with heightened ethanol consumption (Ruiz, Calliari, & Pautassi, 2017). Also, Diaz Heijtz and Castellanos (2006) observed significantly greater insular *c‐fos* expression after the administration of a D1 agonist, in spontaneously hypertensive rats (SHR, an animal model of ADHD) than in Wistar–Kyoto rats. Recently, adolescent rats treated with MPH exhibited reduced activity of the brain‐derived neurotrophic factor and its associated TrkB receptor, at the insular cortex (Wetzell et al., 2014). Several studies propose that reduced activity of the insular cortex plays a key role in the transition from stimulant use to stimulant use disorder (Stewart, Butt, May, Tapert, & Paulus, 2017) and in subsequent relapse (Gowin et al., 2014; Venniro et al., 2017) into stimulant use.

## 2 GENERAL METHODS

### 2.1 Experimental designs

Experiment 1 employed 36 males and 34 females in a 2 (sex) × 2 [drug treatment: 0.0 (vehicle control) or 10.0 mg/kg MPH, once daily for 4 days] factorial design. Experiment 2a employed 72 adolescents, distributed in a 2 (sex) × 2 [treatment: 0.0 (vehicle control) or 4.0 mg/kg AMPH, once daily for 5 days] factorial design. Experiment 2b employed 20 animals, evenly distributed in the design described for Experiment 2a.

### 2.2 Subjects

The rats (strain: Wistar; see Supporting Information for body weight data) were reared at the production vivarium of INIMEC‐CONICET‐Universidad Nacional de Córdoba (UNC; Córdoba, Argentina), a provider of specific, pathogen‐free, animals. They were transferred upon weaning (PD 21) to the animal maintenance room of our laboratory. We did not explicitly measure signs of puberty (e.g., vaginal opening or preputial skinfold separation) in our animals, yet historical observations from our colony indicates that puberty onset in these rats is, in agreement with prior literature (Spear, 2015), between PDs 36–40 for males and about 4–8 days earlier in females. Lighting conditions in both facilities are kept in a 12 hr on—12 off cycle (on at 0700), with a temperature of 20–22°C and a humidity of 45%, approximately. Breeding and experimental procedures followed the Declaration of Helsinki, were approved by the Ministry of Animal Care of INIMEC‐CONICET, and were in accordance with the Guide for the Care and Use of Laboratory Animals of the NIH (National‐Research‐Council, 1996). No more than one rat per litter was assigned to a given group. Unless stated otherwise, the animals were kept in same‐sex groups of four.

### 2.3 Drugs

Methylphenidate (Europharma, Uruguay) and AMPH (Sigma Aldrich, Uruguay) were chronically administered i.p., at a dose of 10.0 or 4.0 mg/kg, respectively (free base; vehicle: 0.9% saline; injection volume: 0.01 ml/g) and expressed as mg of drug administered by a thousand grams (i.e., a kg) of body weight. Drug dosage was chosen based on extensive previous literature [MPH: (Gaytan, Ghelani, Martin, Swann, & Dafny, 1996; Jones & Dafny, 2013); AMPH: (Kameda et al., 2011)]. Our aim was to test whether repeated treatment with these doses, which are commonly used in the literature of animal models of ADHD, would alter subsequent ethanol self‐administration during adolescence and assess potential behavioral and neurochemical correlates of this effect. More in detail, the MPH dose employed is in the upper range of doses employed in animal models of ADHD (Karim, Reyes‐Vazquez, & Dafny, 2017; Venkataraman, Claussen, & Dafny, 2017); and doses equal or lower than 10.0 mg/kg MPH have been found to evoke changes in MPH‐induced motor activity (i.e., behavioral sensitization or tolerance; Chong, Claussen, & Dafny, 2012; Frolov, Reyes‐Vasquez, & Dafny, 2015; Gaytan, Nason, Alagugurusamy, Swann, & Dafny, 2000; Gaytan, Yang, Swann, & Dafny, 2000). Similarly, we employed a dose of AMPH that has been repeatedly employed in the assessment of AMPH‐induced behavioral sensitization and has been shown to yield this phenomenon in adolescent mice (Kameda et al., 2011).

### 2.4 Assessment of drug‐induced motor activity and anxiety response

All animals were given an habituation session (PD26 or 27) to the open field (OF), in which they were administered saline and placed for 10 min in an open field. In the following days, the animals were placed in the dimly lit (circa 50 lux, 60 × 60 × 60 cm) OF, made of Plexiglas and equipped with photocell beams, 40 min postadministration of the corresponding drug treatment (i.e., vehicle, MPH, or AMPH). Beam breaks were transmitted to a monitoring system (ITCOMM, Córdoba, Argentina) that delivered a measure of distance traveled (cm).The rats were withdrawn from the OF after 10 min. The postadministration interval in which motor activity was assessed was chosen based on work by Gaytan et al. (1996).

Anxiety response was assessed via a LDB test routinely used in our laboratory (Wille‐Bille et al., 2017). This assay took place at PD35, after termination of the stimulant treatment, and used a square‐shaped apparatus composed of two sections, one black and lacking illumination (i.e., 0 lux), whereas the other was white and brightly lit (300 lux). Both sections were connected by an opening at floor level. The test lasted for 5 min and began by gently placing the animal in the white sector, opposed to the door opening. The test was videotaped for subsequent measurement of time spent in the white compartment and number of transfer between compartments.

### 2.5 Ethanol intake protocol

We employed a standardized (Wille‐Bille et al., 2017), intermittent‐access intake protocol, composed by three intake sessions beginning at 900AM of PD35, PD37, and PD39 (length: 24 hr per session). At the beginning of each intake session, the animals were weighed and individually transferred to a clean cage lined with pine shavings and equipped with two 100 ml bottles and ad libitum food. They were exposed to two bottles, one contained water and the other contained 5% ethanol. Each bottle was weighed before and after each session to provide an index of fluid intake. The following variables were calculated: grams per kilogram (g/kg) of ethanol ingested, percentage of ethanol intake preference [(ethanol intake/overall liquid intake) × 100], and the overall fluid intake [milliliters of fluid per 100 grams of body weight (ml/100 g)]. We took care of leakage by reading the pre‐ and postsession levels of two bottles, placed in an empty cage. In‐between sessions, the rats were housed in same‐sex couples and had unrestricted access to food and water.

The rationale for using 5% ethanol is that this concentration is similar to that of the beverages usually consumed by adolescents. A study (Pinsky, Zaleski, Laranjeira, & Caetano, 2010) found that more than half of the ethanol ingested by adolescents is derived from beer, a drink containing 3%–8% ethanol. Sixty‐seven percent of adolescents aged 18–20 years who incurred in binge drinking in the United States also reported beer as their beverage of choice (Naimi, Brewer, Miller, Okoro, & Mehrotra, 2007). It also has been reported that ethanol‐naïve, adolescent Wistar, rats drank very little ethanol when given at concentrations ≥6%. In previous studies, we overcame this by exposing the adolescents to substantial water deprivation or by mixing the drug with sucrose (Ponce, Pautassi, Spear, & Molina, 2011). In this study, however, we deemed better to avoid the caveats associated with the caloric surplus of sucrose and the stress induced by dehydration.

### 2.6 Assessment of dopamine, serotonin, and norepinephrine

We followed a procedure similar to that described in Ruiz et al. (2017). Briefly, the animals were killed by decapitation. The encephalous was quickly obtained, and the insular cortex was dissected following the technique described by Aleksandrov and Fedorova (2003). The tissue was kept frozen at −80°C until further processing. The samples were then weighed and sonicated in 1 ml of perchloric acid (0.1 mol/L). The resulting solution was centrifuged to obtain a supernatant. Those samples were injected into an HPLC system (PM‐80 BAS, West Lafayette, IN, USA) equipped with a C18 column and a LC‐4C BAS electrochemical detector. The flow was kept at 1 ml/min, and the mobile phase was composed by 0.15 mol/L acetic acid, 0.6 mol/L octyl sulfate of sodium, acetonitrile (4%), and tetrahydrofuran (1.6%, PH 3). Data are expressed as ng/g. These measurements were conducted at the Neurochemistry Laboratory of the Instituto de Investigaciones Biológicas Clemente Estable (Montevideo, Uruguay).

### 2.7 Specific methods of Experiment 1

All the rats (*n* = 70, 36 males and 34 females) received daily administration of MPH or vehicle, during four days (PDs 28–31).

Thirty‐eight of these subjects (*n* = 10 in the male groups treated with MPH or vehicle, *n* = 9 in the female groups) were assessed for motor activity after each administration and subsequently, on PDs 35, 37, and 39, were tested for ethanol intake. The remaining 32 rats (*n* = 8 in each group) were tested for anxiety response in the LDB test on PD35. Of these rats tested in the LDB, 22 (11 male, 11 female, *n* = 7 in MPH‐treated groups, *n* = 4 in vehicle‐treated groups) were sampled on PD36 for DA concentration at the insular cortex.

### 2.8 Specific methods of Experiment 2

The 72 male and female adolescents used in Experiment 2a were treated daily with AMPH or vehicle during 5 days (PDs 27–31). On PD33, they were all challenged with a lower dose [2.0 mg/kg, based on work by Adriani, Chiarotti, and Laviola (1998)] than that used during the induction phase. Forty of these animals (*n* = 10 per group) were assessed for motor activity after each administration and subsequently, on PDs 35, 37, and 39, were tested for ethanol intake. The remaining 32 rats (8 in each group) received AMPH or vehicle, yet were not tested for motor activity in the OF nor for ethanol intake. Instead, they were tested in a LDB test on PD35.

Experiment 2b employed 10 adolescent males and 10 adolescent females. Five animals of each sex were treated daily with AMPH and the rest were given vehicle. On PD36, these animals were submitted to the assays for measurement of catecholamine levels. In addition to DA, norepinephrine and 5‐HT (transmitters known to be significantly involved in the regulation of ethanol intake) were also measured.

### 2.9 Data analysis

Body weight (across experiments, recorded prior to each motor activity session or prior to the intake test sessions), distance traveled in the OF (cm), absolute ethanol intake (g/kg), percent ethanol preference, and overall fluid intake (ml/100 g of body weight) during the intake sessions were analyzed by repeated‐measures analyses of variance (ANOVA). Depending on the experiment, sex, drug treatment during the repeated treatment, and drug treatment at the challenge served as between‐subjects factors. Session was the within‐subjects factor. Across experiments, the ANOVAs for body weight scores indicated that males were heavier than females and that this pattern was significantly similar across MPH‐, AMPH‐, or vehicle‐treated rats. Body weights (mean ± *SEM*) across experiments are provided as Supporting Information in the online version of the article.

Each behavior measured in the LDB (Experiment 1 and 2a) and the levels of DA, norepinephrine, and 5‐HT were independently analyzed using independent factorial ANOVAs, with sex and drug treatment as independent factors.

Tukey's post hoc test (α = 0.05) was used to analyze significant main effects and interactions comprising “between” factors, and Cohen's partial eta squared (η²*p*) was used to calculate effect sizes. Planned comparisons were used to analyze significant between factor × within factor interactions. The rationale for this distinction is that there is a lack of appropriate post hoc tests to analyze interactions that involve both between‐subjects and within‐subjects factors (Winer, Brown, & Michels, 1991). Please note that, given the difficulty of illustrating significant main effects or significant interactions that span several conditions and groups, some of the significant differences have not been presented in the figures (via pounds or other signs). In those instances, however, a description of these significant differences can be found in each figure legend.

In animals given AMPH or MPH‐repeated treatment we conducted, separately for each sex, Pearson correlations between ethanol intake scores (g/kg ingested each testing day and mean g/kg ingested across days) and the percentage of change in distance traveled in the last day of treatment relative to the first day of treatment.

All statistical analyses were conducted using the software Statistica 6.0 (STATISTICA, RRID:SCR_014213).

## 3 RESULTS

### 3.1 Experiment 1

**Figure 1:**
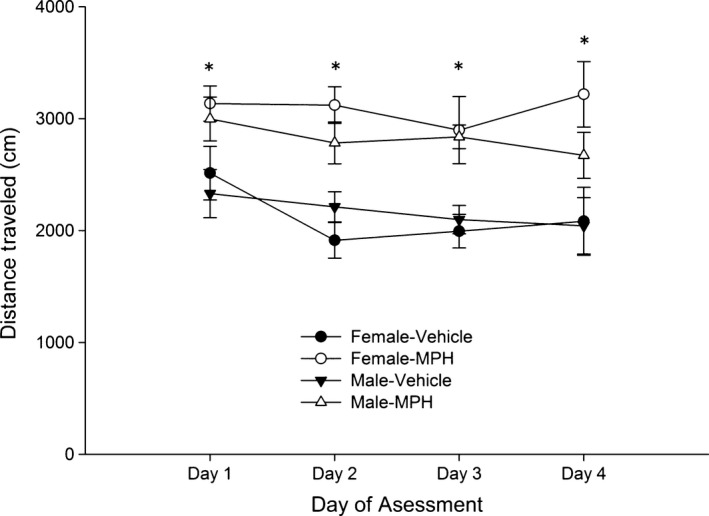
Distance traveled (in centimeters) in the open field test in Experiment 1 in male and female Wistar rats as a function of days of assessment (1–4, which correspond to postnatal days 28–31) and methylphenidate (MPH) dose administered before the test [0.0 (vehicle) or 10.0 mg/kg). The statistical analysis revealed that MPH‐treated rats exhibited greater distance traveled than vehicle‐treated counterparts, and this stimulant effect of MPH was similar in males and females and across days of testing. These significant differences are indicated by the asterisks. The data are expressed as mean ± *SEM*

The analysis for distance traveled across sessions revealed a significant main effect of treatment, *F*_1,34_ = 28.40, *p *<* *.001, η²*p* = .46. As shown in Figure 1, MPH‐treated rats exhibited greater distance traveled than vehicle‐treated counterparts and this stimulant effect of MPH was similar in males and females and across days of testing.

**Figure 2:**
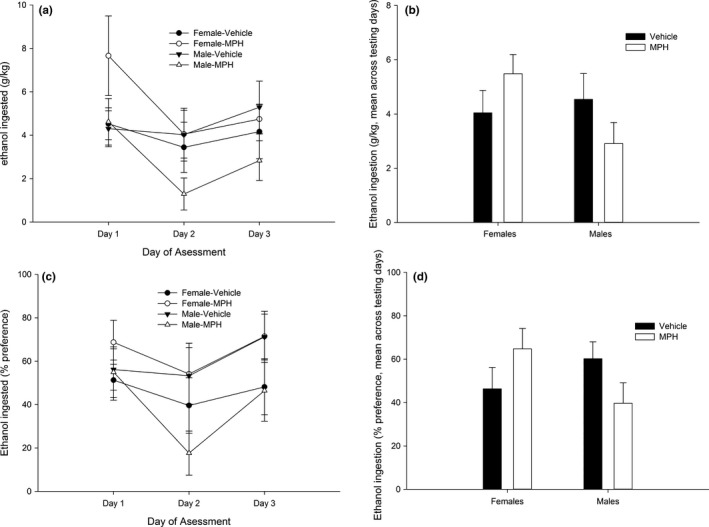
(a–d) Mean ethanol intake (g/kg) (a) and percent preference (c) in male and female Wistar rats as a function of methylphenidate (MPH) dose administered on postnatal days 28–31, during sessions (i.e., days of assessment) 1, 2, and 3 of the intake protocol (Experiment 1). Two‐bottle intake sessions (5% ethanol vs. plain water) were conducted on Monday, Wednesday, and Friday (session length: 24 hr), beginning on postnatal day 35 (PD35) and ending on PD40. The data are expressed as mean ± *SEM*. (b, d) Same as (a, c), collapsed across the three testing sessions. The statistical analysis revealed no significant difference between MPH‐control and vehicle‐control counterparts, at any day of test. Please refer to the text for a full account of the statistical analyses

The ANOVA for ethanol intake (g/kg, upper panel of Figure 2) yielded a significant interaction between drug treatment and session, *F*_2,68_ = 3.18, *p *<* *.05, η²*p* = .19. The post hoc tests conducted within each treatment indicated that MPH‐treated, but not vehicle‐treated, rats exhibited a significant decrease in g/kg ethanol ingested from the first to the second intake session. No significant between‐group difference was observed, however, when contrasting, via the post hoc tests, MPH‐control and vehicle‐control counterparts, at any day of testing.

The ANOVA for percent preference only yielded a significant main effect of day, with significantly lower ethanol consumption in the second than in the first or third session. These results are depicted in the lower section of Figure 2. The ANOVA for overall liquid consumption scores (see descriptive statistics in Supporting Information) revealed a significant main effect of session: *F*_2,68_ = 5.54, *p *<* *.01, η²*p* = .14. The post hoc tests revealed greater overall liquid intake in the first than in the second or third sessions.

The Pearson correlations indicated the lack of significant associations between the percentage of change in MPH‐induced distance traveled (i.e., PD31 scores relative to PD28 scores) and ethanol intake scores.

The ANOVA for number of transfers between compartments (LDB test) and for time spent in the white compartment (see Table 1) revealed significant main effect of sex (*F*_1,28_ = 7.29, *p *<* *.05, η²*p* = .20 and *F*_1,28_ = 6.32, *p *<* *.05, η²*p* = .64, for each variable, respectively). The post hoc tests indicated that both behaviors were significantly greater in females than in males. The ANOVA of the dopamine concentration at the insula (see Table 1) yielded no significant main effect or significant interactions.

**Table 1:** Behavioral responses in a 5‐min light–dark box test [LDB: Time (s) spent in the white compartment and frequency of transfers between compartments], and dopamine, serotonin, and norepinephrine concentrations (ng/g tissue) in adolescent, male, and female, rats as a function of the pharmacological treatment received during postnatal days 28–32 [0.0 or 10.0 mg/kg methylphenidate (MPH, Experiment 1) and 0.0 or 4.0 mg/kg amphetamine (AMPH, Experiment 2). The data are presented as mean ± *SEM*

	Females				Males			
	10.0 mg/kg MMPH	0.0 mg/kg MPH	4.0 mg/kg AMPH	0.0 mg/kg AMPH	10.0 mg/kg MPH	0.0 mg/kg MPH	4.0 mg/kg AMPH	0.0 mg/kg AMPH
LDB Time spent in white (segs)	44.6 ± 12.9	55.1 ± 13.3	82.8 ± 33.8	174.0 ± 47.0	9.2 ± 3.7	38.1 ± 8.8	35.8 ± 7.2	45.8 ± 6.8
LDB Number of transfers	10.2 ± 2.7	11.6 ± 2.9	3.7 ± 1.0	2.7 ± 1.0	2.6 ± 0.8	7.5 ± 1.5	5.0 ± 1.1	3.8 ± 0.6
Dopamine concentrations (ng/g of tissue)	1336.0 ± 400.4	515.7 ± 241.3	1083.5 ± 359.6	1884.4 ± 290.1	640.1 ± 70.0	593.3 ± 82.2	1154.2 ± 148	1854.8 ± 482.1
Serotonin concentrations (ng/g of tissue)	—	—	456.6 ± 100.3	585.4 ± 55.2	—	—	474.6 ± 31.1	546.7 ± 87.3
Norepinephrine concentrations (ng/g of tissue)	—	—	5.0 ± 0.5	285.9 ± 38.6	—	—	342.2 ± 34.3	274.2 ± 33.9

### 3.2 Experiment 2

**Figure 3:**
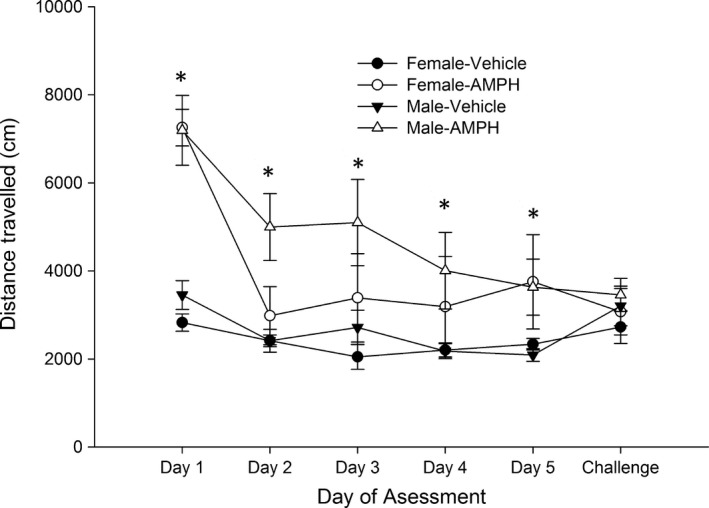
Distance traveled (in centimeters) in the open field test in Experiment 2a in male and female Wistar rats as a function of days of assessment (1–5, which correspond to postnatal days 27, 28, 29, 30, and 31 and a challenge day on postnatal day 33) and amphetamine dose (AMPH) dose administered before each test [0.0 (vehicle) or 4.0 mg/kg). At the challenge, all the rats were given 2.0 AMPH. The statistical analysis revealed that rats treated with AMPH, regardless sex, displayed significantly higher locomotor activity than those treated with vehicle during in days 1, 2, 3, 4, and 5, but not during the challenge. These significant differences are indicated by the asterisks. The data are expressed as mean ± *SEM*

The analysis of distance traveled (see Figure 3) revealed significant main effects of treatment and days (*F*_1,35_ = 26.11, *p *<* *.001, η²*p* = .42 and *F*_5,175_ = 13.02, *p *<* *.001, η²*p* = .27), and a significant treatment × days interaction (*F*_5,175_ = 9.86, *p *<* *.001, η²*p* = .22). The post hoc tests indicated that distance traveled was significantly greater in AMPH versus vehicle‐treated rats in all but the last testing session.

**Figure 4:**
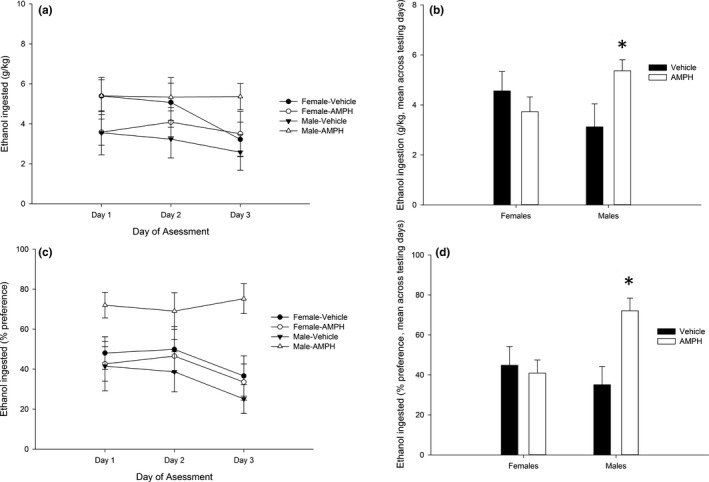
(a–d) Mean ethanol intake (g/kg) (a) and percent preference (c) in male and female Wistar rats as a function of amphetamine dose (AMPH) dose administered at postnatal days 27–31 [0.0 (vehicle) or 4.0 mg/kg)], during sessions (i.e., days of assessment) 1, 2, and 3 of the intake protocol (Exp. 2a). Two‐bottle intake sessions (5% ethanol vs. plain water) were conducted on Monday, Wednesday, and Friday (session length: 24 hr), beginning on postnatal day 35 (PD35) and ending on PD40. The data are expressed as mean ± *SEM*. (b, d) Same as (a, c), collapsed across the three testing sessions. The statistical analysis revealed that, when compared to the pertinent vehicle‐treated same‐sex control, ethanol intake and preference were significantly enhanced across sessions in male, but not in female, rats treated with AMPH during the repeated treatment of postnatal days 27–31. This significant difference is indicated by the asterisks. Please refer to the text for a full account of the statistical analyses. The data are expressed as mean ± *SEM*

The ANOVA for gram per kilogram of ethanol ingested yielded a significant main effect of AMPH treatment (*F*_1,35_ = 4.19, *p *<* *.05, η²*p* = .11) and a significant interaction between AMPH treatment and sex (*F*_1,35_ = 4.60, *p *<* *.05, η²*p* = .12). The ANOVA for percent preference also revealed a significant AMPH treatment × sex interaction (*F*_1,35_ = 6.43, *p *<* *.05, η²*p* = .15). The post hoc tests conducted after the significant interaction yielded similar results: AMPH‐treated male, but not female, rats exhibited significantly greater ethanol intake (g/kg ingested) and significantly greater ethanol percent preference than vehicle‐treated counterparts. These results are in Figure 4.

The ANOVA for overall liquid consumption scores (data not shown) yielded a significant main effect of session: *F*_2,66_ = 38.50, *p *<* *.01, η²*p* = .54. The post hoc tests revealed greater overall consumption, which was not affected by AMPH treatment or sex, in the second than in the first or third sessions. There was no significant correlation between the percentage of change in AMPH‐induced distance traveled (PD32 relative to PD28) and ethanol intake scores.

The ANOVA for number of transfers between compartments (LDB test, see Table 1) only yielded a significant main effect of sex (*F*_1,28_ = 8.87, *p *<* *.01, η²*p* = .24), with the post hoc tests revealing significantly greater frequency of this behavior in females than in males. The ANOVA for time spent in the white compartment did not reveal significant min effects nor significant interactions.

The ANOVA for 5‐HT levels did not reveal significant main effects or significant interactions. The ANOVA for norepinephrine levels indicated a significant sex × treatment interaction (*F*_1,16_ = 31.90, *p *<* *.01, η²*p* = .66). The post hoc tests indicated that females, but not males, treated with AMPH exhibited reduced norepinephrine levels than same‐sex, vehicle‐treated controls. The ANOVA for DA levels, in turn, revealed a significant main effect of treatment (*F*_1,16_ = 4.81, *p *<* *.05, η²*p* = .23). The post hoc tests revealed AMPH treatment significantly reduced insular levels of DA in both. Mean ± *SEM* (ng/ml) DA, 5‐HT, and norepinephrine levels can be found in Table 1.

## 4 DISCUSSION

The main new finding of the present study is that treatment with AMPH, but not with MPH, enhanced ethanol intake during late adolescence, in male Wistar rats. More in detail, the males that had been exposed to AMPH had a mean average ethanol intake slightly below 5.5 g kg^−1^ 24 hr^−1^, whereas control males drank around 3.0 g kg^−1^ 24 hr^−1^. AMPH pre‐exposed males also exhibited, when compared to peer counterparts, a twofold increase in preference for ethanol (70% vs. 35%, respectively) versus water. The significant, AMPH‐induced, increase in ethanol intake was specific for ethanol and did not generalize to the total quantity of fluids consumed. On the other hand, drinking of ethanol in the females was not affected by AMPH treatment and MPH was mostly devoid of a modulatory effect upon ethanol ingestion in either sex.

There are few studies that assessed the effects of stimulant treatment during adolescence on subsequent ethanol consumption, and to our knowledge, this is the first that conducted both events (stimulant treatment and measures of ethanol intake) within the time frame of adolescence. The biggest novelty of the present work is that it shows that even a very short treatment with AMPH during early adolescence can render male adolescents susceptible for greater ethanol intake. This is important because an early age of onset of ethanol consumption or an early age of first drunkenness rank among the best predictors of subsequent development of ethanol dependence (Pilatti, Caneto, Garimaldi, Vera Bdel, & Pautassi, 2014). As a comparison, other work (Gill et al., 2014) found heightened drinking of sweetened ethanol after MPH treatment in young adult rats, yet this effect was observed only in rats given environmental enrichment housing conditions and after three weeks of treatment with the stimulant (8 mg kg^−1^ day^−1^ for 21 days, delivered via an osmotic pump). Also important is a study (Crowley, Cody, Davis, Lovinger, & Mateo, 2014) in which male C57Bl/6J mice were exposed to MPH (3–6 mg kg^−1^ day^−1^, dissolved in their drinking water bottle) throughout adolescence (PDs 30–60). This treatment resulted in increased DA clearance at adulthood and a blunted response to the motor‐activating effects of ethanol. Despite these neural and behavioral effects, no significant differences in ethanol drinking (tested across a wide range of concentrations: 3%–20%) were observed between MPH‐treated and control mice (Crowley et al., 2014).

A comparison can also be made between the present set of results and a study conducted, in adult female rats (Fahlke, Hansen, Engel, & Hard, 1994). These researchers gave AMPH or its vehicle daily, in an escalating scheme (from 1 to 9 mg kg^−1^ injection^−1^) and across a lengthy period (5‐week). AMPH‐treated rats exhibited enhanced intake of ethanol when tested three months after termination of the drug dosing. This work and our study concur in suggesting that repeated AMPH treatment can enhance subsequent ethanol intake. A main difference between the studies is that we failed to observe an AMPH‐induced increase in ethanol intake in females. Major sources of explanation for this discrepant finding are the age tested, and the length and dose AMPH exposure.

What mechanisms led to the increased, AMPH‐induced, ethanol intake found in Experiment 2? One possibility is that this repeated drug treatment yielded alterations in anxiety response or in risk‐taking patterns, which promoted the ingestion of ethanol. These traits have been long considered phenotypes associated with propensity for ethanol intake. We have recently observed, for instance, that rats selectively bred for high ethanol intake during adolescence significantly avoided the white, potentially dangerous, chamber of a light–dark box (Fernandez et al., 2017); and in other study, we found that male rats reared under environmental enrichment exhibited both enhanced ethanol intake and behaviors indicative of greater risk‐taking, when compared to counterparts reared under standard conditions (Berardo, Fabio, & Pautassi, 2016). In the present study, however, the patterns of exploration of the light–dark box were similar in AMPH, MPH, and vehicle‐treated rats. There was a sex effect, with the males displaying more inborn anxiety than the females, yet this was independent of the drug treatment.

Other possibility is that repeated AMPH treatment favors the development of behavioral sensitization, which in turn is associated with greater risk for exacerbated ethanol seeking and intake (Camarini & Pautassi, 2016). Support for this hypothesis comes from a study in which psychostimulant users exhibited heightened heart rate response to an ethanol challenge (Brunelle, Barrett, & Pihl, 2006). We analyzed sensitivity to the acute and chronic motor‐activating effects of AMPH and MPH. Across the experiments, both AMPH and MPH exerted reliable acute motor stimulant effects. Yet, there was no indication of a gradual increase in this response. Similarly, the challenge test conducted in Experiment 2 failed to reveal a modulatory effect of the chronic treatment on the acute effect of the drug.

A first implication of the motor activity results is that there seems to be dissociation between the ability of these stimulant drugs to induce behavioral sensitization and their effects upon ethanol consumption: AMPH did not induce behavioral sensitization yet it significantly altered ethanol consumption in Experiment 2. Also, the lack of AMPH‐induced or MPH‐induced sensitization in our weaned, adolescent rats contrasts with results of studies with preweanling rats. Duke, O'Neal, and McDougall (1997) observed significant behavioral sensitization in rats given 2.5 mg/kg AMPH, twice daily, during PDs 17–20. Other studies applied only one daily dose of AMPH (1.0, 2.5 or 5.0 mg/kg) or MPH (5.0, 10.0 or 15.0 or 20.0 mg/kg) during PDs 17–20 (McDougall, Duke, Bolanos, & Crawford, 1994) or PDs 16–21 (McDougall, Collins, Karper, Watson, & Crawford, 1999) and reported behavioral sensitization when tested 24 or 48 hr after the induction phase.

When the previous studies and the present study are put together, it seems that AMPH or MPH can induce motor behavioral sensitization during early ontogeny but not during adolescence. This is consistent with some, but not all, previous studies. A study (Kozanian, Gutierrez, Mohd‐Yusof, & McDougall, 2012) found, albeit using stimulants other than AMPH or MPH, a sensitized response to the repeated administration of methamphetamine or cocaine in preweanling, but not in adolescent, rats. Conversely, adolescent but not adult mice exhibited greater motor activity to a challenge dose of AMPH (2.0 mg/kg), after a history of exposure to 10.0 mg/kg AMPH (Adriani et al., 1998). It has been suggested that the mechanisms responsible for the expression of long‐term sensitization to psychostimulants are just concluding maturation by the fourth week of rats’ postnatal life (Tirelli, Laviola, & Adriani, 2003), which could explain the variability in the reported results.

Administration of AMPH in adult rats often induces robust and long‐lasting behavioral sensitization, which can also be observed a few days after the induction. Kuczenski and Segal (2001), for instance, gave adult Sprague–Dawley rats 0.1 or 0.25 mg/kg of AMPH, twice daily for 5 days. Treatment with the latter dose resulted, 4 days later, in an augmented response to a 0.5 g/kg AMPH dose. The findings of other studies suggest, however, that our adolescents could have shown behavioral sensitization if a shorter protocol had been used. Specifically, Kameda et al. (2011) found greater behavioral sensitization to AMPH in adolescent versus adult mice, as induced by a single injection of 4.0 mg/kg AMPH.

The effects of repeated MPH administration have not been consistent, with behavioral sensitization reported by some (Gaytan, al‐Rahim, Swann, & Dafny, 1997; Kuczenski & Segal, 2001; Sripada, Gaytan, Al‐rahim, Swann, & Dafny, 1998) but not by other (Crawford, McDougall, Meier, Collins, & Watson, 1998; Izenwasser et al., 1999) studies. Most of these studies, however, employed a prolonged pretreatment or induction phase and were conducted in adult subjects only. The studies that assessed the effects of exposure to MPH during adolescence have also produced inconsistent results. MPH treatment during adolescence reduced subsequent CPP by cocaine (Andersen, Arvanitogiannis, Pliakas, LeBlanc, & Carlezon, 2002) yet enhanced cocaine‐induced motor activity and self‐administration (Brandon, Marinelli, Baker, & White, 2001). In another study, adolescent male Wistar rats that had been given MPH (orally from PD 27–33) did not exhibit behavioral sensitization when challenged with either MPH (1.0 or 10.0 mg/kg) or nicotine (0.4 mg/kg; Justo, Carneiro‐de‐Oliveira, Delucia, Aizenstein, & Planeta, 2010).

The heightened ethanol intake, found in the males after AMPH treatment (Experiment 2a), was associated with a significant decrease in insular levels of DA (Experiment 2b). MPH, on the other hand, did not significantly alter insular levels of DA, and MPH‐treated rats exhibited a significant decrease in absolute ethanol intake from the first to the second intake session (Experiment 1). These differences could relate to the fact that AMPH blocks the dopamine transporter (DAT) and the vesicular monoamine transporter, whereas MPH only blocks DAT (Kuczenski & Segal, 1997). Is it possible to link the dopaminergic deficit to the greater ethanol intake? Ethanol intake in rats can be driven by DA deficits in the nucleus accumbens (Feltmann, Fredriksson, Wirf, Schilstrom, & Steensland, 2016), and ethanol craving in humans is associated with low levels of DA synthesis in the dorsal striatum (Heinz et al., 2005). It also worth noting that the rationale for focusing our measurements at the insula was that adolescent rats with low insular levels of DA exhibited depression‐like behavior and increased ethanol intake (Ruiz et al., 2017). It is, however, difficult to draw a direct relationship between the dopaminergic deficit and the greater ethanol intake found in the males of Experiment 2a. On one hand, the studies by Feltmann et al. and Heinz et al. were conducted in rats and humans with a long history of ethanol exposure. Also, in Experiment 2b, the AMPH‐induced reduction in insular DA was also found in females, yet they did not show alterations in ethanol intake.

The results of Experiment 2b, nonetheless, are consistent with a plethora of studies indicating that chronic treatment with AMPH can alter the functioning of the dopaminergic system. DA release in the lateral septum was found to be reduced in adult rats daily injected AMPH (2.5 mg/kg for 14 days; Renard, Sotomayor‐Zarate, Blanco, & Gysling, 2014), and earlier work indicated DA depletion and loss of receptors in the dorsal striatum (Krasnova et al., 2001; Wagner, Ricaurte, Johanson, Schuster, & Seiden, 1980) and in the olfactory bulb (Atianjoh, Ladenheim, Krasnova, & Cadet, 2008), after repeated AMPH treatment. It is interesting to remark that termination of a 6‐day AMPH exposure to rats (5.0 or 10.0 mg kg^−1^ day^−1^) has been associated with depressive‐like behavior and deficits in brain reward function, as indicated by increased intracranial self‐stimulation (Cryan et al., 2003). Also, repeated exposure to AMPH or MPH has been used to induce psychotic‐like states (Frey et al., 2006). We did not measure these variables, yet we cannot discard that similar alterations may have emerged in our study. Also important is that the experimental design did not include subjects outside of the adolescent period, and thus, it cannot be concluded that the pattern found is an age‐specific, developmental effect.

The present results should be considered in the context of important limitations. A single MPH dose and AMPH dose were used across experiments. This precludes assessing graded, dose‐response effects and, moreover, the doses employed are in the upper range of those commonly employed in the literature and significantly beyond those dispensed orally to children affected by ADHD [for a discussion on which combination of dose, route of administration and interval between administration better mimics in rodents the treatment used in humans, please see Kuczenski and Segal (2001)]. There are also between‐experiments methodological changes that complicate the comparisons and interpretation of the results.

Another important limitation is that the MPH and AMPH doses employed were not equated for their level of drug‐induced motor stimulation. Moreover, the dose of amphetamine use in Experiment 2 induces stereotypy (Salisbury & Wolgin, 1985; Wolgin, 2012), yet the method of analyzing motor stimulation employed (photocell beam breaks) does not allow measurement of stereotypy. It is possible, thus, that the measures of amphetamine‐induced locomotion were confounded, and probably reduced, by this drug increasing stereotypy. Moreover, a very limited monoamine analysis was conducted (i.e., only the insular levels were addressed). The rationale was that studies from our laboratory (Ruiz et al., 2017) and from others [e.g., (Diaz Heijtz & Castellanos, 2006) have suggested an association between neural activity or DA levels at this structure and ethanol intake or stimulant‐induced behavioral activity.

Despite these limitations, the main contribution of the study is that a short treatment with AMPH during adolescence, subthreshold in terms of inducing behavioral sensitization, can significantly alter dopamine levels in the insula in male and females, and enhances ethanol intake and preference in males. Chronic exposure to MPH did not ostensibly affect these outcomes.

## CONFLICT OF INTEREST

None declared.
