# Supplementary material for: Amphetamine, but not methylphenidate, increases ethanol intake in adolescent male, but not in female, rats
Source: Brain Behav. 2018 Feb 19;8(4):e00939. doi: 10.1002/brb3.939 (PMC5893334; doi:10.1002/brb3.939)
Supplement: Supplementary file 1 [file BRB3-8-e00939-s001.doc]

**Supplementary information**

**Supplementary information 1.** Body weight (g, first number of each line) and overall liquid consumption scores (ml/100 g of body weight, second number of table lines 7-9, presented between brackets and in *italics*) in adolescent rats, males and females, during open field and two-bottle intakes sessions conducted on Experiment 1. The animals were administered metilphenidate (MPH, 10.0 mg/kg) or vehicle (VEH; i.e., 0.0 mg/kg MPH) on postnatal days 28 to 31. The data are presented as mean ± SEM.

|  | Females | | Males | |
| --- | --- | --- | --- | --- |
|  | MPH group | VEH group | MPH group | VEH group |
| Open field session 1 | 98.6±3.6 | 96.6±2.9 | 116.5±4.6 | 121.7±3.7 |
| Open field session 2 | 104.2±3.2 | 106.8±2.2 | 125.1±4.6 | 128.7±3.5 |
| Open field session 3 | 117.2±3.1 | 117.2±2.2 | 138.6±3.9 | 144.8± 3.1 |
| Open field session 4 | 120.1±3.3 | 119.8±3.7 | 139.7±5.7 | 146.7±5.3 |
| Intake session 1 | 129.0±3.3  (*27.8±6.0*) | 127.0±3.3  (*23.9±2.1*) | 156.0±4.8  (*20.7±1.4*) | 162.0±3.2  (*19.2±1.4*) |
| Intake session 2 | 134.3±4.1  (*18.8±0.8*) | 132.8±2.9  (*21.0±1.1*) | 164.8±5.1  (*19.9±2.0*) | 172.0±2.8  (*18.2±1.9*) |
| Intake session 3 | 142.1±4.3  (*16.7±1.0*) | 136.1±2.6  (*22.5±1.7*) | 177.0±5.6  (*15.6±0.8*) | 181.8±3.3  (*18.3±2.5*) |

**Supplementary information 2.** Body weight (g) in adolescent rats during open field test, challenge and subsequent two-bottle intakes sessions conducted on Experiment 2. The data are presented as mean ± SEM.

|  | Females rats | | Males rats | |
| --- | --- | --- | --- | --- |
|  | AMPH group | VEH group | AMPH group | VEH group |
| Open field session 1 | 81.4 ± 1.5 | 82.6 ± 1.5 | 91.5 ± 2.7 | 91.0 ± 1.7 |
| Open field session 2 | 84.4 ± 2.2 | 77.2 ± 4.1 | 97.5 ± 2.7 | 99.0 ± 2.5 |
| Open field session 3 | 89.1 ± 2.8 | 81.8 ± 4.5 | 97.8 ± 6.1 | 103.0 ± 4.1 |
| Open field session 4 | 98.1 ± 1.6 | 91.8 ± 3.7 | 112.1 ± 3.4 | 104.7 ± 5.1 |
| Open field session 5 | 91.8 ± 3.5 | 95.6 ± 4.5 | 117.7 ± 3.8 | 107.1 ± 6.6 |
| Challenge session | 113.4 ± 3.3 | 109.5 ± 4.2 | 138.3 ± 3.9 | 134.0 ± 5.3 |
| Intake session 1 | 117.9 ± 3.5 | 116. ± 4.7 | 143.8 ± 3.9 | 140.4 ± 5.5 |
| Intake session 2 | 129.3 ± 3.3 | 128.3 ± 4.4 | 159.1 ± 4.4 | 157.7 ± 6.1 |
| Intake session 3 | 138.3 ± 4.4 | 139.8 ± 4.9 | 180.0 ± 4.2 | 177.8 ± 6.7 |
